# The Essentials of Experimental Physiology

**Published:** 1899-03

**Authors:** 


**The Essentials of Experimental Physiology.** By T. G. Brodie, M.D. Pp. xiv., 231. London: Longmans, Green, and Co. 1898.

For some time past we have had most excellent guides for laboratory work in histology and chemical physiology, by Schäfer and Halliburton respectively. Dr. Brodie's is the much wished for companion volume, and will, we think, have the same success. It is written on lines similar to the others—containing a clear account of the main experiments which should be done in such a course, and of the deductions which may be drawn from them. It is admirably done, and will, we hope, be much used.

